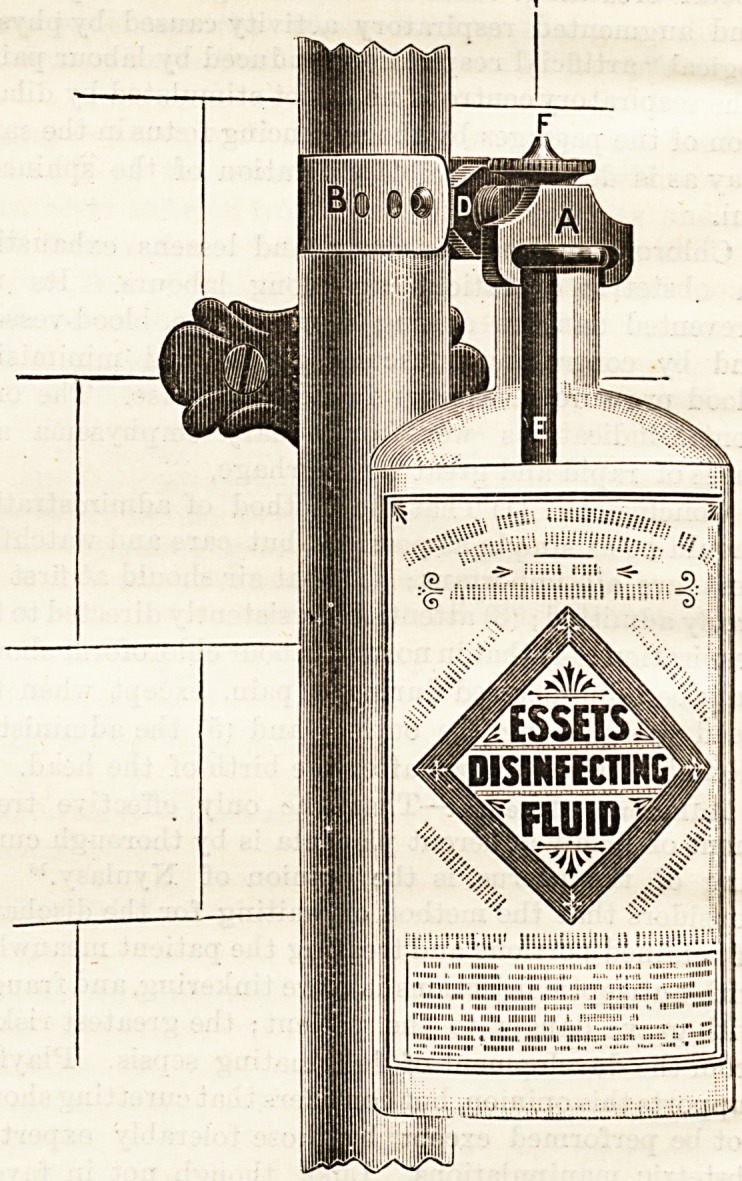# New Appliances and Things Medical

**Published:** 1897-05-01

**Authors:** 


### NEW APPLIANCES AND THINGS MEDICAL.

### NOURRY'S IODINATED WINE.

(Agents F. Comar and Son, 64, Holborn Viaduct, E.C.)

For nearly ten years this wine has been before the public, but the success which has attended its use in France, where it originated, has taken some time to travel to this country. This is all the more remarkable, as the medical profession has been constantly on the look out for a convenient way of administering iodine. Those who continue the use of iodide of potassium have long wished for a reliable substitute, and many have thought to overcome the difficulties attending the constant dosing with the potassium salt by prescribing a mixture of the iodides of sodium, potassium, and ammonium. In Nourry's Iodinated Wine we have a comparatively tasteless compound of iodine and tannin dissolved in a Malaga wine, making a very palatable form of administering the drug, which is not objected to by the most fastidious of patients. When a drug can be conveniently administered in the form of a wine, the patient does not look upon the regular dosage as inconvenient, and in this way the due and proper effect of the iodine is assured. There is an abundance of clinical information showing that iodine in the iodotannin condition exerts the usual therapeutic effect of the drug, and if the amount of iodine per wine-glassful can be guaranteed, we see no reason why it should not be frequently prescribed in this form. Our special analysis of the samples submitted to us reveals that the wine has an extract amounting to 32·15 per cent., and that it contains no free iodine, as starch fails to give any reaction. The quantity of alcohol present was 8·71 per cent., absolute alcohol by weight, and the acidity, both volatile and fixed, after making due allowance for the tannin, indicate a genuine wine. The determination of the iodine is not an easy matter, although it is readily shown to be present by qualitative tests. By distilling off the alcohol under reduced pressure, and then liberating the iodine by means of sulphuric acid and a nitrite, we were enabled to distil off the whole of the iodine, and then titrate the liberated iodine by "Thio" in the well-known way. The amount claimed by the proprietors is 3·3 grams per litre, or 0·05 gram per tablespoon. Our estimation shows only 2·0 grams per litre, and we notice that one of our contemporaries some time ago also found that the amount of iodine was less than stated. It is to be regretted that the proprietors do not take care to ensure a regular and definite amount of the active ingredient in each bottle, and we suggest that they should state definitely on each bottle the weight of iodine in grams per litre and the number of grains of iodine per fluid ounce. We object strongly to the indefinite tablespoonful, and if grams per litre and grains per fluid ounce are both stated, no one could be misled as to the exact strength of the wine. As iodinated wine differs from many other medicated wines in being only desirable to use for short periods under the direct advice of a medical man, we hope the proprietors will see their way to adopting our suggestion, and thus standardise what is otherwise an indefinite preparation involving a certain amount of uncertainty in its use.

### THE "UNIQUE" AUTOMATIC FLUSH DISINFECTOR.

(A. Granville and Co., 102, High Street, Battersea, S.W.)

This is an apparatus to be attached to the flush pipe of a water closet, and is so arranged that a certain quantity of disinfecting fluid is discharged along with each flush. The chief points in favour of the use of this apparatus are that it can be easily fixed, that any fluid disinfectant can be used, that the fluid is drawn direct from the bottle, that the amount used can be regulated, and that when the bottle has become empty it can easily be refilled or replaced. It may be asked, why should a "disinfector" be used at all, and if used is it likely to disinfect? Now, we do not think that any householder would be likely to use sufficient of any disinfectant to render a two-gallon flush germicidal, but nevertheless these "disinfectors" serve a useful purpose. However perfectly the flush may empty a closet pan, there are occasions when the water which remains is somewhat soiled, and still more frequently does it happen that the water in the trap is not quite clean. Hence arises the "fusty" odour of so many closets, and there can be no doubt that this can be largely prevented by the addition of a little "disinfectant" to each flush.

## Figures and Tables

**Figure f1:**